# The Institutional Worker

**Published:** 1912-02-24

**Authors:** 


THE
### INSTITUTIONAL WORKER
**Being a Special Supplement to "The Hospital"**

### Editor's Notices.

**Contributions:**

Contributions should be written, or preferably typed, on one side of the paper only, and all articles sent in are accepted upon the distinct understanding that they are forwarded to The Hospital only.

The Editor cannot undertake to return MSS. not used, but when a stamped directed envelope is enclosed the MSS. may be returned if a special request is made.

Accepted articles and paragraphs of news will be paid for after publication at the scale rate.

**Address:**

To prevent delay all contributions and letters on editorial business must be addressed exclusively to the Editor, "The Hospital," 29 Southampton Street, Strand, London, W.C. It is important that this regulation shall be strictly observed.

**Correspondence:**

Correspondence on all subjects is invited. The name and address of correspondents must be given as a guarantee of good faith, but not necessarily for publication.

**Special Articles:**

Special articles are invited, and questions, inquiries, and paragraphs upon all matters relating to the work, administration, and management of general, special, mental, fever, cottage and convalescent hospitals, sanatoria, homes, institutions, societies and organisations for the treatment or care of the sick, injured and dependents of all classes. Every matter affecting the interests and welfare of the staff of all grades working in these institutions will receive special consideration.

**Administrative Medicine, Research and Items of News:**

Special terms will be paid for approved articles on subjects in this wide field by experts who have made some section of it their special study and interest. We wish to give prominence to every movement tending to advance accuracy of diagnosis, efficiency in treatment, and the development of modern methods to eradicate disease and promote the welfare of the suffering.

**A Bureau of Information:**

We invite inquiries and applications for information and help in providing for that numerous class of sufferers who require special care or house-room which they cannot obtain in their own homes or secure for themselves. We cannot, however, prescribe or recommend practitioners.

**Books for Review:**

Publishers are particularly requested to send advance proofs of any new books of importance whenever possible, as the Editor has made arrangements to publish immediate reviews on a new plan.

**Photographs, Plans, Blocks, and Illustrations:**

It is requested that wherever possible MSS. may be accompanied by illustrations in any of the above forms. The name of the sender and of the article to which it belongs should be written on the back of each photograph, drawing, or block for purposes of identification.

**Local Papers:**

Newspapers containing reports or news paragraphs should be marked and addressed to the Sub-Editor.

**The Coupon System:**

A coupon will be found at the bottom of the third inside page of the cover each week. This coupon must be attached to every question or inquiry to which an answer is desired in The Hospital.

### Manager's Notices.

Letters relating to the publication, sale, and advertising departments of The Hospital must be addressed to the Manager, "The Hospital," The Hospital Building, 28 and 29 Southampton Street, Strand, London, W.C.

Subscriptions which may begin at any time, are payable in advance. Cheques and Post-Office orders, crossed London County and Westminster Bank, Covent Garden branch, should be made payable to the Manager of The Hospital and addressed to him at The Hospital Building, 28 and 29 Southampton Street, Strand, London, W.C.

**Advertisements:**

All advertisements for The Hospital must reach the Manager not later than Wednesday morning in each week. For scale of charges see pages v and 2.

**Institution Authorities.**

The economical management of an Institution depends very greatly upon purchasing supplies in the cheapest market.

It is often impossible locally to secure the highest quality at the lowest prices.

In consequence, a section will be reserved in this part of the paper for announcements of Institutions inviting Tenders for Contracts.

This will enable Authorities of Institutions to secure tenders for supplies from all over the country, and ensure purchases at the lowest possible prices consistent with quality.

The charge for Tenders is 6s. per inch, and for this small outlay many pounds may be saved on supplies of all descriptions.

Special Rates will be quoted for those institutions which agree to send all their vacancy, contract, and public notices to The Hospital each year, so as to make it a file of such announcements for ready reference.

**Classified Advertisements:**

All small advertisements will be classified, for this section of the paper is widely read and of special interest. We wish to encourage those wanting appointments who are attracted to institutional work, and therefore offer them the reduced rate of *twenty-one words and under for* 1s., and *for every ten and part of ten words beyond*, 6d.

This form may be used for small prepaid advertisements, and when filled up should be posted to the

*Manager*, The Hospital,
28 and 29 Southampton Street,
Strand, W.C.

| | | | |
|---|---|---|---|
| Appointments Wanted, 21 words | ... | 1s. | 0d. |
| Appointments Vacant, 21 words | ... | 2s. | 6d. |

### OFFICIAL APPOINTMENTS VACANT.

### Poor-Law Vacancies.

ASSISTANT CLERK (£90), Southwark Union. Mr. S. Wood, C. to G., Ufford Street, Blackfriars, S.E. Feb. 29.

ASSISTANT LAUNDRESS (£27), St. Pancras Guardians. Matron of the Workhouse, King's Road, N.W.

ASSISTANT MALE NURSE (£30), Hackney Union. Medical Superintendent of the Infirmary, High Street, Homerton, N.E.

ASSISTANT MASTER (£40), Romford Union. Mr. C. Bloomfield, C. to G., Romford. Feb. 26.

ASSISTANT NURSE (£25), Evesham Union. Mr. E. H. Wadams, C. to G., Evesham. Feb. 25.

ASSISTANT NURSE (£25), Glanford Brigg Union. F. C. Hett, C. to G., 11 Bigby Street, Brigg. Feb. 29.

ASSISTANT NURSE (£22), Bermondsey Guardians. E. Pitts Fenton, C. to G., 283 Tooley Street, S.E. March 4.

ASSISTANT NURSE (£27), Burton-on-Trent Union. C. F. Chamberlain, C. to G., Union Offices, Burton-on-Trent. March 6.

ASSISTANT NURSES (2) (£28 each), West London District Schools. Mr. F. G. Beeching, Clerk to the Managers, Ashford, Middlesex.

ASSISTANT NURSE (£18), Holborn Union. Matron of the Workhouse, Mitcham, Surrey.

ASSISTANT NURSES (£26), Whitehaven Union. Mr. E. B. Croasdell, C. to G., Whitehaven. Feb. 26.

CHARGE FEMALE IMBECILE ATTENDANT (£30), Wigan Union. Mr. H. G. Ackerly, C. to G., Wigan. Feb. 26.

CHARGE NURSE (£30), Norwich Guardians. E. J. W. Huggins, C. to G., St. Andrew's Street, Norwich.

CHARGE NURSE (£28), Holborn Union. Matron of the Workhouse, 1A Shepherdess Walk, N.

CHARGE NURSE (£40), Brighton Guardians. B. Burfield, C. to G., Parochial Offices, Brighton. March 5.

CHARGE NURSE (£32), Huddersfield Union. E. A. Rigby, C. to G., Union Offices, Ramsden Street, Huddersfield. March 7.

CHARGE NURSES (2) (£31 to £35, according to qualifications), East Preston Union. Mr. A. Shelley, C. to G., Littlehampton. Feb. 26.

COOK, ETC. (£18), Newbury Union. Mr. S. V. Pinniger, C. to G., Newbury. March 4.

FEMALE ATTENDANT (£25), Lambeth Guardians. Matron of the Parish School, Elder Road, West Norwood, S.E.

FEMALE ATTENDANT (£23), Islington Guardians. Matron of the Workhouse, Cornwallis Road, Upper Holloway, N.

FEMALE BATH ATTENDANT, ETC. (£25), Islington Guardians. Master of the Workhouse, Cornwallis Road, Upper Holloway, N.

FEMALE EPILEPTIC WARD ATTENDANT (£20), Holborn Union. Matron of the Workhouse, Mitcham, Surrey.

FOSTER-MOTHER (£26), Stamford Union. Mr. R. M. English, C. to G., Stamford. Feb. 24.

FOSTER-MOTHER (£23), Wells Union. Mr. A. G. Russ, C. to G., Wells, Somerset. Feb. 27.

FOSTER-MOTHER (£25), Worksop Union. Mr. J. S. Whall, C. to G., Worksop. Feb. 27.

HEAD NURSE (£30), Redruth Union. Mr. T. Peter, C. to G., Redruth. Feb. 29.

HOME SISTER (£30), Stockport Union. The Matron of the Workhouse, Stockport.

INFANTS' NURSE (£30), Bridgend and Cowbridge Union. Mr. R. H. Cox, C. to G., Bridgend, Glam. Feb. 24.

JUNIOR ASSISTANT-NURSE (£15), Malling Union. F. J. Allison, C. to G., West Malling, Kent.

LABOUR MASTERS (2) (£30 each), Islington Guardians. Master of the Workhouse, Cornwallis Road, Upper Holloway, N.

LABOUR MISTRESS, ETC. (£30), Brentford Union. Matron of the Workhouse, Isleworth, W.

LAUNDRESS (£30), Wigan Union. Mr. H. G. Ackerley, C. to G., Wigan. Feb. 24.

LAUNDRYMAID (£18), St. George's Union. Matron of the Infirmary, Fulham Road, S.W.

MALE ATTENDANTS (£20), Salford Union. Mr. F. Townson, C. to G., Salford. March 5.

MALE NURSE (£30), King's Lynn Union. Mr. R. C. Coulson, C. to G., King's Lynn. Feb. 27.

MASTER AND MATRON (£50 and £30), Brecknock Union. Mr. M. F. Thomas, C. to G., Brecon. March 6.

MASTER'S CLERK (£37 10s.), Farnham Union. Master of the Workhouse, Farnham.

MASTER'S CLERK (£40), Leigh Union. Mr. A. H. Hayward, C. to G., Leigh. Feb. 28.

MATRON (£150), Birmingham Guardians. C. Fletcher, C. to G., Parish Offices, Edmund Street, Birmingham. Feb. 29.

MIDWIFERY PUPIL, Dewsbury Union. C. P. Pickersgill, C. to G., Union Offices, Wellington Street, Dewsbury. Feb. 29.

NEEDLEMISTRESS, ETC. (£25), Brentford Union. Matron of the Workhouse, Isleworth, W.

NIGHT ATTENDANT FOR MOTHERS' NURSERY (£25), West Ham Union. Mr. T. Smith, C. to G., Leytonstone, N.E. Feb. 28.

NURSE (£30), Merthyr Tydfil Union. Mr. F. T. James, C. to G., Merthyr Tydfil. March 1.

NURSE, ETC. (£30), Midhurst Union. Mr. J. M. Furneaux, C. to G., Midhurst. March 2.

NURSE (£27 10s.), Stockbridge Union. Matron of the Workhouse, Stockbridge.

NURSE (£25), Worksop Union. Mr. J. S. Whall, C. to G., Worksop. Feb. 27.

NURSE (£30), Aylsham Union. Mr. H. J. Gidney, C. to G., Aylsham, Norfolk. Feb. 26.

NURSE (£25), Richmond Union, Surrey. P. Umney, C. to G., Parkshot, Richmond. March 6.

NURSE (£35), Plymouth Guardians. W. H. Davy, C. to G., Greenbank Road, Plymouth. Feb. 27.

NURSE (£20), St. Pancras Guardians. J. E. P. Hall, C. to G., Town Hall, Pancras Road, N.W.

NURSES (£32), Leigh Union. A. H. Hayward, C. to G., Union Offices, Leigh, Lancs. Feb. 28.

NURSES (2) (£30 each), Gateshead Union. Mr. G. Craighill, C. to G., Gateshead. Feb. 24.

NURSES' ASSISTANT (£16), Blandford Union. Mr. S. Traill, C. to G., Blandford.

NURSE-ATTENDANT (£24), Islington Guardians. Matron of the Workhouse, St. John's Road, Upper Holloway, N.

PROBATIONER NURSE (£10), Kidderminster Union. F. E. Burcher, C. to G., Bank Buildings, Kidderminster. March 4.

RELIEVING OFFICERS (2) (£150), Islington Guardians. Mr. E. Davey, C. to G., St. John's Road, Upper Holloway, N. Feb. 26.

SECOND ASSISTANT LAUNDRESS (£24), West Ham Union. Mr. T. Smith, C. to G., Leytonstone, N.E. Feb. 28.

STAFF NURSES (£25), Salford Union. Mr. F. Townson, C. to G., Salford. March 5.

STAFF NURSES (£26), Doncaster Union. H. M. Marshall, C. to G., Union Offices, High Street, Doncaster.

SUPERINTENDENT NURSE (£35), Haslingden Union. Mr. J. H. Sinkinson, C. to G., Rawtenstall. Feb. 28.

SUPERINTENDENT OF LAUNDRY (£35), Norwich Guardians. Mr. E. J. W. Huggins, C. to G., Norwich. Feb. 27.

WARDSWOMAN (£18), West London District Schools. Mr. F. G. Beeching, Clerk to the Managers, Ashford, Middlesex.

### Institutional and Administrative Vacancies.

ASSISTANT MATRON (£36), Bedford County Hospital. B. Wadmore, Secretary.

MATRON (£45), Bangor (Co. Down) Hospital. Hon. Secretary, Town Hall, Bangor, Co. Down. March 2.

MATRON (£100), Royal Halifax Infirmary. O. Webster, Secretary. March 6.

OPHTHALMIC SISTER (£28), Northampton General Hospital. Matron.

STAFF NURSE (£25), N. Staffs Infirmary, Stoke-on-Trent. Matron.

### Municipal Vacancies.

ASSISTANT CLERK (£70), Aylesbury U.D.C. Mr. P. A. Wright, Clerk to Town Council, Aylesbury. March 1.

ASSISTANT INSPECTOR OF NUISANCES (£2 weekly), Conway R.D.C. Mr. T. E. Parry, Clerk to the Council, Conway. Feb. 28.

CANVASSER (£3 weekly), Sunderland Corporation Electricity Works. Mr. A. S. Blackman, General Manager, Electricity Works, Sunderland.

CHARGE NURSE (£35), Cuckfield R.D.C. Isolation Hospital. Mr. C. H. Waugh, Clerk to the Council, Hayward's Heath. Feb. 26.

DRAUGHTSMAN (£3 weekly), Sunderland Corporation Electricity Works. Mr. A. S. Blackman, General Manager, Electricity Works, Sunderland.

FEMALE SANITARY INSPECTOR (£2 2s. weekly), Birkenhead Borough. J. Fearnley, Town Clerk, Town Hall, Birkenhead. Feb. 26.

INSPECTOR OF NUISANCES AND SURVEYOR (£75), Llanidloes T.C. Mr. A. Davies, Town Clerk, Llanidloes. March 9.

JUNIOR CLERKS (£70 minimum), M.A.B. The Clerk to the Board, Embankment, E.C. Feb. 27.

LADY HEALTH VISITORS (2) (£78), Manchester Corporation. T. Hudson, Town Clerk, Town Hall, Manchester. March 2.

LAUNDRESS (£26), West Ham T.C. Mr. F. E. Hilleary, Town Clerk, Stratford, E.

LEDGER CLERK (£91), Rhondda U.D.C. The Accountant, Council Offices, Pentre, Rhondda. Feb. 24.

MATRON (£50), Aberdare U.D.C. Isolation Hospital. Mr. T. Phillips, Clerk to the Council, Aberdare. Feb. 28.

MATRON (£80), Bournemouth Corporation. H. Ashling, Town Clerk, Municipal Offices, Bournemouth. March 6.

NURSE (£25), Aberdare U.D.C. Isolation Hospital. Medical Officer of Health, 43 High Street, Aberdare. March 2.

NURSE (Health Visitor) (£80), Southampton T.C. The Medical Officer of Health, Southampton. Feb. 26.

NURSES (2) (£32 10s.), Lanchester Joint Hospital Board. Mr. W. H. Ritson, Clerk to the Board, Lanchester. Feb. 29.

NURSE-MATRON (£50), Pontardawe Hospital Committee. Mr. W. Lewis, Clerk to the Committee, Pontardawe, Glam. Feb. 24.

